# Virtual simulated international placements as an innovation for internationalisation in undergraduate programs: a mixed methods study

**DOI:** 10.1186/s12909-023-04260-x

**Published:** 2023-04-19

**Authors:** Amanda K. Edgar, James A. Armitage, Nadeeka Arambewela-Colley, Luke X. Chong, Anuradha Narayanan

**Affiliations:** 1 School of Medicine (Optometry), Faculty of Health, Deakin University, 75 Pigdons Road, Waurn Ponds, 3216 Australia; 2 Partnerships and Engagement, Office of the Executive Dean, Faculty of Health, Deakin University, 221 Burwood Highway, Burwood, 3125 Australia; 3 Elite School of Optometry, Medical Research Foundation, Chennai, India

**Keywords:** Virtual simulation, Optometric education, Clinical reasoning, Virtual placements, Teaching and learning innovations, Online international mobility

## Abstract

**Background:**

Inherent features in virtual simulation could be utilised to deliver collaborative global education that is inclusive, accessible, and valued by students and facilitators. The aim of this study was to evaluate the impact of the International Eyecare Community (IEC) platform’s virtual simulated international placements (VSIP) in optometric education.

**Methods:**

An international, multi-center, cross-sectional mixed methods study with Deakin University, Australia, and the Elite School of Optometry, India, was used to evaluate the impact of VSIP in the IEC using pre-existing deidentified data collected from teaching and learning activities within the optometry course curriculum. Data on students and facilitators perceptions of the VSIP were collected through deidentified transcripts from focus group discussions. The data were interpreted using descriptive statistics and qualitative analysis using constant comparison for thematic analysis.

**Results:**

A total of 64 out of 167 student participants completed survey responses (39%) and 46 out of 167 (28%) completed self-reflective inventories. Focus groups with 6 student participants and 6 facilitator participants were recorded and analysed. Student participants reported the IEC was relevant (98% agreement) and motivated them to apply theoretical knowledge to a clinical context (97% agreement). The themes identified through qualitative analysis were: factors inherent to the virtual simulation that enabled learning through VSIP, the VSIP supported cognitive apprenticeship, VSIP enabled clinical learning for optometric education, VSIP’ role in cross-cultural professional identity development in optometry students.

**Conclusion:**

The study found that the VSIP platform helped to motivate students to learn and improve their clinical skills. The VSIP was considered a potential supplement to physical clinical placements and could revolutionize global optometric education by offering co-learning across cultures.

**Supplementary Information:**

The online version contains supplementary material available at 10.1186/s12909-023-04260-x.

## Background

It is important for optometry students to develop clinical competency by practising the application of clinical knowledge and skills in a variety of patient cases and presentations before they graduate and work independently as a qualified practitioner. The traditional methods in teaching that facilitate this are learning activities such as clinical placements where students are typically posted within the professional community [1, 2]. The learning value of clinical placements can be constrained by poor student engagement, limited number of placement sites compared to the demand from a growing number of students, and inequity in the quality and diversity of learning experiences with an unpredictable variety of patient encounters [1, 3]. In 2020–2022, rolling lockdowns in response to the COVID-19 pandemic reduced access to clinical training facilities and amplified these drawbacks. One strategy to address some of the limitations and challenges encountered due to the pandemic was integrating virtual simulation into the curriculum.

The International Eyecare Community (IEC) was created with the purpose to incorporate the inherent advantages of virtual simulation and deliver collaborative global education by offering flexible, diverse, personalised, accessible and equal learning opportunities [4, 5]. This platform was not created to replace face-to-face teaching; instead, it offered an additional resource for teaching and facilitated the application of clinical knowledge and complex clinical skills using pedagogy inherent to traditional clinical placements that involved self-reflection and repetitive practice [6]. The platform leverages off learners’ familiarisation with modern digital environments such as learning management systems and internet locations that support their learning [5]. The activities in the IEC platform included an asynchronous self-guided case workup and synchronous virtual simulated international placements (VSIP) where optometry students, optometrists and ophthalmologists from Deakin University and Sankara Nethralaya/Elite School of Optometry worked through an authentic clinical case together and discussed key findings and considerations associated with that case.

The aim of this study was to evaluate the impact of IEC VSIP in optometric education. The purpose of this study was to answer the following research questions:

1. Do online virtual international placements in the IEC engage optometry students and motivate them to study?
2. Do optometry students value the VSIP resources provided?
3. Do VSIP in optometry produce improved optometry students’ skills and ability?
4. Do VSIP in optometry offer training in cognitive complexity that is meaningful?

The results will potentially have a positive impact on curriculum development in optometric education in Australia and India relating to continued innovation and internationalization of optometric education in the context of increasing globalized and digital connected world.

## Methods

### Design

This is an international, multi-center, cross-sectional mixed methods study. It aims to evaluate the impact of VSIP using pre-existing deidentified data collected from teaching and learning activities within the optometry course curriculum and deidentified transcripts from focus group discussions. The VSIP were scheduled to take place over a period of five weeks. During this time, there were a total of twenty-one synchronous learning activities. Each student had the opportunity to self-select and participate in two out of the twenty-one sessions. Student experiences were evaluated as part of a quality assurance activity using a voluntary and anonymous survey instrument at the completion of the fifth week. Pre-existing scores from the Diagnostic Thinking Inventory for Optometry (DTI-O) before and after the learning activity were included in the study [7]. After the 5^th^ week, all students and facilitators were invited to participate in focus group discussions with two to four students or two to four facilitators.

### Ethical considerations

Ethics approval (Deakin HEAG-H30_2012; Sankara Nethralaya 1150–2023-P) was obtained in accordance with all local guidelines and regulations at each institution from the relevant administrators with written informed consent obtained after all aspects of the study were explained. Informed consent was obtained from all subjects and/or their legal guardian(s) for publication of identifying information/images in an online open-access publication. All research conducted was performed in accordance with the principles stated in the Declaration of Helsinki.

### Setting and population

This study was conducted in the optometry program, within the School of Medicine at Deakin University, Australia and the Elite School of Optometry, India. All students and facilitators that participated in the VSIP program in the IEC for 2020 were invited to participate in this study on a voluntary basis.

#### Students

Students from Deakin University School of Medicine, Faculty of Health were from the optometry department within the second year of the program of the Bachelor of Vision Science/Master of Optometry course (10-trimester double degree, completed in 3.5 years). For this stage of the curriculum, students participate in observing patient care in a clinical setting but do not actively participate in clinical care. Students had undertaken 3 trimesters of foundational units, followed by a problem-based learning framework horizontally and vertically integrated with clinical skills and other preclinical training, prior to experiencing the VSIP.

Students from the undergraduate (4-year, 8 semester) and postgraduate (2-year, 4 semester) optometry program at Elite School of Optometry were also part of the study. From their third-year students participate in observing patient care in a clinical setting and participate in clinical care by their fourth year of undergraduate studies. These students have undergone 3 semesters of foundational coursework, core optometry coursework, clinical skills training and supervised clinical rotations by the end of their degree. The total number of students from the undergraduate and post graduate studies at 75.

#### Facilitators

Facilitators for the session were trained to encourage students to co-construct knowledge in a student-centred environment. These facilitators had a special interest in the content area of the session they were involved in, and their role was to use tactics that support metacognitive questioning, push students for ideas, help students develop hypothesis and create learning issues. Four optometrists and eleven ophthalmologists working at Sankara Nethralaya, a tertiary eye care hospital in Chennai, India, were engaged as facilitators on this project. These facilitators worked alongside five practicing optometrists that were also Deakin University academics who were involved as facilitators from Australia.

#### A global learning platform

**Fig. 1:**
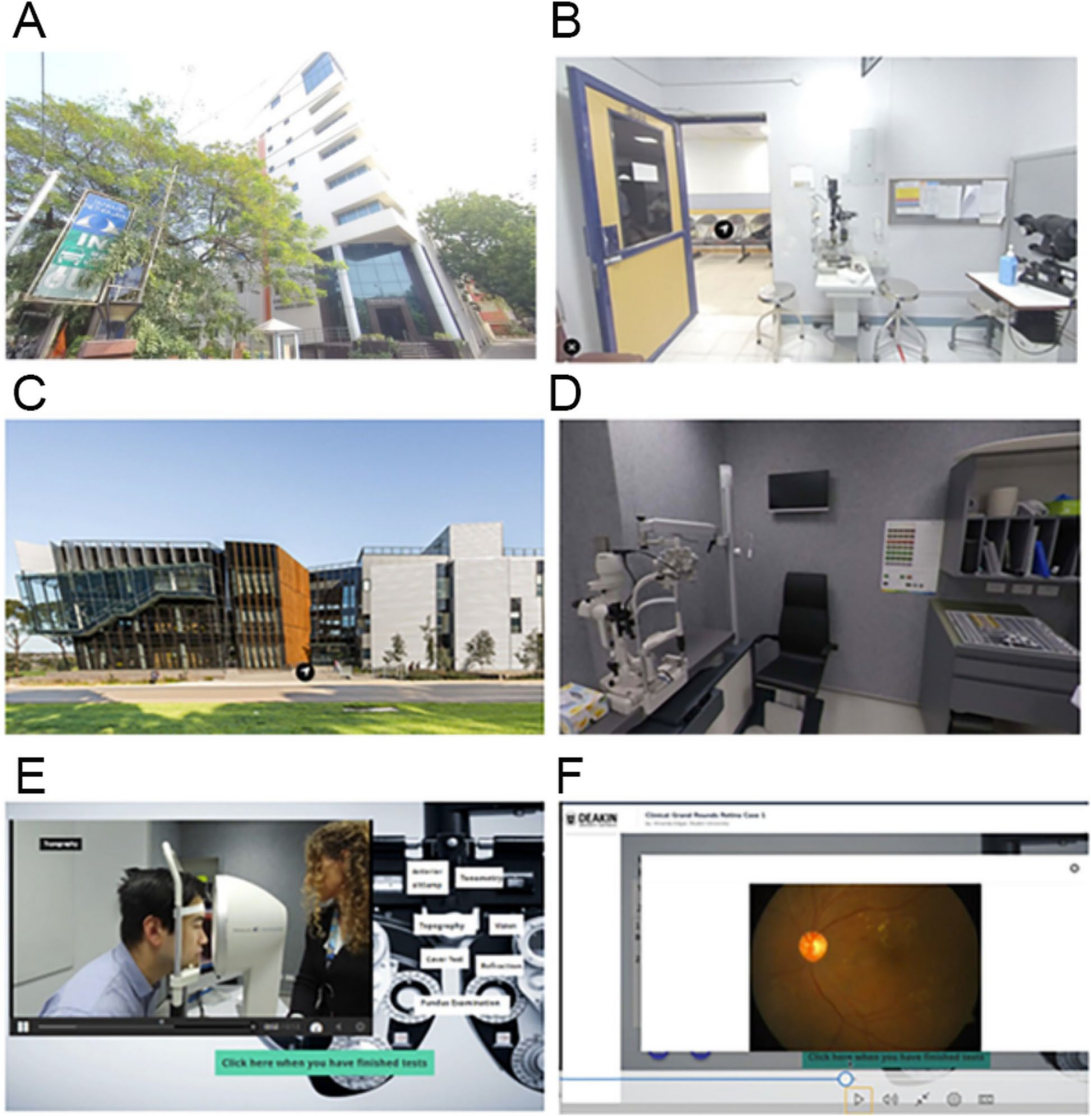
Screen shots from the user view of the International Eyecare Community. **A** Users’ view from their personal device of the entrance to the virtual Sankara Nethralya eye hospital. **B** Users’ view moving through the virtual optometry consultation rooms in India. **C** Users’ view from their personal device of the entrance to the virtual Deakin Collaborative Eye Care Clinic. **D** Users’ view moving through the virtual optometry consultation rooms in Australia. **E** Users’ view of the simulated authentic clinical case. **F** Users’ view of the recorded virtual placement embedded into the virtual hospital locations

The virtual simulation activities were housed in a global learning platform called the IEC using a virtual representation of a hopital in India, Sankara Nethralaya, and an optometry teaching clinic in Australia, Deakin Collaborative Eye Care Clinic (Fig. 1). This web-based platform was accessible for students and facilitators using a device with internet connection. To incorporate the IEC into teaching and learning VSIP were created.

#### The VSIP

**Fig. 2:**
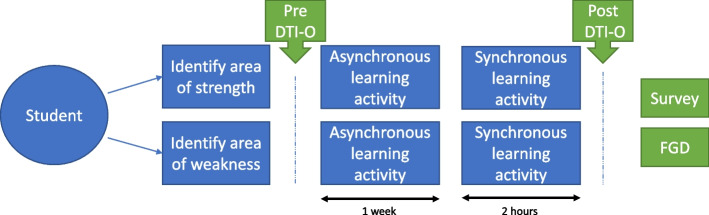
This schematic representation of a student’s workflow through the VSIP. Abbreviations: VSIP, virtual simulated international placements

The VSIP involved students completing an independent asynchronous learning activity followed by synchronous learning activity. These were delivered in January – March 2021. At this time, due to COVID-19, international travel was prohibited, and physical international mobility was not possible. The VSIP and study data collection timepoints are outlined in Fig. 2. Each student was allocated to two VSIP sessions. To determine what session they would attend all students completed a self-reflective survey (Additional file 1) prior to the VSIP commencing. This asked them to choose one area of optometry practice they would like to master with the autonomy to select an area they were interested in (area of strength) and one area they felt they needed the most improvement (area of weakness). These options that were available to select were chosen by optometry academics who were also practicing clinicians in both countries. This ensured that these areas represented a wide diversity of optometry practice in both countries and would nurture rich discussions through international collaborative case-based activities. Examples of the eleven topics included are binocular vision, glaucoma, emergency, low vision and paediatrics.

Students completed an asynchronous online learning activity one week before the scheduled synchronous learning activity in the IEC based on the area of optometry that they had self-selected. Prior to commencing each asynchronous activity students were asked to complete the DTI-O in an online form to reflect on their self-perceived diagnostic reasoning ability in the allocated area of optometry. The asynchronous online learning activities were developed using authentic clinical cases from the Sankara Nethralaya hospital based on pedagogy in consultation with content experts and health education experts from both countries. The asynchronous online learning activities were designed to step students through a process of information gathering that is specific to the clinical case and require them to prioritize, eliminate, where necessary, and integrate information to combat diagnostic uncertainty and make an evidence-based decision that replicates an experienced optometry practitioner’s decision [8]. The openness of the task made students solely responsible for their time and environment management.

The synchronous learning activity was scheduled so that students could meet through the IEC platform in groups of 15–20 from ESO and Deakin with academics, optometrists and/or ophthalmologists to work through the online learning activity. As students repeated the task with peers, the aim was to increase motivation with the addition of peer support and collaboration as well as inter-professional communication skills, cultural awareness, and clinical reasoning. In each session the student group received feedback by content experts from both institutions. Following completion of the VSIP the students repeated the DTI-O. All students who participated in the VSIP were invited to participate in a focus group to capture the meaningfulness of the task and opinions of authenticity, realism, inter-professional communication skills, cultural awareness, and clinical reasoning.

### Instrument

A survey instrument (Additional file 2) with 21-items was used to evaluate the teaching activities. The survey instrument was distributed as a link using an online software platform (Qualtrics, Provo, UT) and was posted to the announcement board on course websites as a voluntary and anonymous activity available to be accessed by all students. Survey completion required approximately 15 min. Face validity was determined by researchers using their experience in optometric education and international mobility by reviewing the concepts of the questions. The group comprised one international coordinator (mobility), undertaking a PhD in medical anthropology; four experienced optometric educators and practicing optometrists, two from India and two from Australia. Students rated the value of the VSIP to outline concepts such as the significance of virtual simulation in optometric education and self-reflected improvement in clinical competency skills. The survey asked participants to rank how closely they agreed with a statement using a 5-point Likert scale, 1 (strongly disagree) to 5 (strongly agree) and discuss their experiences using open-ended questions.

### Focus group

Focus group discussions were conducted for the student participants and facilitator participants separately to gain an in-depth understanding about the experience with the VSIP [9]. A guide (Additional file 3) with probes was prepared by AE, AN, JA and NC after discussion and mutual agreement on the questions. The focus group discussions were held in four sessions: Elite School of Optometry student participants; Deakin University student participants; Sankara Nethralaya facilitator participants; and Deakin University facilitator participants. Researchers from Australia ran focus groups for facilitators and students from India and vice versa to ensure that no researchers had prior relationships with participants in the focus group discussions. The discussions were continued until there was saturation of information considering the responses from the survey as well [10].

### DTI-O scores

The DTI-O was used to measure self-reflected diagnostic reasoning skills in participants. This is a 41-item inventory and uses a 6-point scale that alternates positive responses left and right to avoid complacency of the user. The inventory has two subdomains, 21 items for Flexibility in Thinking (FT) and 20 items for Structured Memory (SM). Flexibility in thinking focuses on the skill of moving between different enquiry processes and structured memory relates to the ability to process possessed knowledge to solve clinical cases [7]. A higher score on the DTI-O represents greater diagnostic reasoning skills with a maximum score of 246 (126 in FT and 120 in SM). The DTI-O has demonstrated ability to detect the difference between abilities in diagnostic reasoning in a validity study and was chosen to reflect the student’s level of diagnostic reasoning proficiency at a set point in time [7].

### Data analysis

Dichotomous data was acquired for non-parametric quantitative analysis using the survey results. These responses were interpreted by percentage of agreement and disagreement and, where appropriate, descriptive statistics using Statistical Package for Social Sciences (SPSS) (version 21). Tables 1, 2 and 3 highlight the responses to questions 7–10 in percentage for each question. Statistical analysis of the DTI-O results was performed on all items and pre and post activity results were compared using a Wilcoxon Signed-Ranks test.

Thematic analysis was completed on focus group discussion transcripts and open-ended survey questions using a phenomenological approach with codes forming hierarchical nodes and reduced to define emergent themes. Two independent coders (AN and AE) used ten participants surveys for thematic content and these coders developed a draft set of themes so they could be reviewed and mapped to thematic domains. AN and AE then coded 10% of response before reviewing any discrepancies in major themes to ensure there was alignment of the codes with the research questions. AN and AE independently coded the remaining data using the coding structure. Responses were then coded by one independent coder (JA) and then reviewed with AN and AE discussing areas of overlapping themes, disagreement, merger of codes and exclusion of content until consensus was reached. All representative comments by participants on major themes were chosen and agreed upon by all co-authors and then extracted for illustration of themes. Sample size was based on convenience sample of all students and facilitators that participated in VSIP.

## Results

Of 167 students (Deakin *n* = 92 and ESO *n* = 75) that were given the option to complete the online survey, we received 64 responses (39%) with 19 from Deakin University and 45 from Elite School of Optometry. Of these participants *n* = 22 were in their 2^nd^ year, *n* = 22 3^rd^ year, *n* = 12 4^th^ year, *n* = 4 5^th^ year, *n* = 4 6^th^ year and all responses were used in the analysis. The responses are representative of the cohort given the high response rate. In addition, following the online survey focus groups discussions with 6 students and 6 facilitators (Deakin *n* = 2 and ESO *n* = 4) were held to generate rich data from in-depth interviews until data saturation was achieved. Of the 167 students 46 (28%) completed the DTI-O before and after the VSIP.

The final findings of thematic analysis are presented alongside the quantitative results to support triangulation of qualitative insights. There were 4 themes and their subthemes, identified from an initial 28 codes once data saturation was achieved. Quotes are reported verbatim from participants in italics with clarifying insertions in square brackets and an identification number. The quantitative results are presented as a percentage of the responses.

### Theme 1: factors inherent to the virtual simulation that enabled learning through VSIP

Through data analysis researchers explored the factors inherent to virtual simulations that enabled learning in the VSIP. Realism is a factor inherent to virtual simulation that leads to successful user engagement, described as the ability to mirror reality and suspend disbelief [11]. As part of the researchers’ exploration, there was a concerted effort to understand if the learning strategies were as real as possible. The realism of the experience in the VSIP was expressed by participants to produce meaningfulness, and they acknowledged that what they were learning was valuable to them as a student because it was real.

*“We learn optometry in a real way… rather than learning it theoretically.”* (P37).

*“The virtual simulated experience made me imagine being in an optometrist place and examining the patient”* (P17).

This sub-theme, realism, was further supported by quantitative responses in Table 1.

**Table 1:** Student perception of the realism of the virtual simulated international placements

Question:	Very Inaccurate n (%)	Inaccurate n (%)	Unsure n (%)	Accurate n (%)	Very Accurate n (%)
*How accurately does the virtual simulated environment represent your perception of an optometry setting?*	3 (4%)	2 (3%)	12 (19%)	44 (69%)	4 (5%)

The student participants expanded on this to explain that the realism enabled them to test their understanding and clinical decisions before being in the clinical environment, which they felt increases learning confidence and clinical competence when performing tasks in physical clinical settings.

*“… because we are using real life scenarios, we know how to think and how to process… to make a better decision when while dealing in real clinics.”* (P5).

Participants also discussed that the VSIP is an opportunity to provide an equitable and inclusive experience. Facilitators and student participants reflected that the VSIP provides a opportunity for all students, including students who otherwise may not have had the capacity (for a variety of reasons) to engage in an international learning experience during their Optometric studies.

*"…some students might have had faced barriers… actually attending India in person and its sort of more an equitable experience."* (P7).

*“Virtual platform helped everyone equally.”* (P4).

The participants expressed that an inherent feature was that the VSIP were a customised learning space where learning happens based on the individuals demands on time and their chosen learning.

*"A virtual way of learning helps the people to learn from anywhere at any time."* (P29).

The student participants expressed preference for the learning activity compared to traditional lectures with 32 (50%) participants preferring virtual simulation some of the time, 24 (38%) most of the time and 5 (8%) all the time. Table 2 outlines the participants perception of the relevance of the VSIP and how this increased their motivation for learning.

**Table 2:** Student perception of the VSIP

Question: *The virtual simulation…*	Strongly Disagree n (%)	Disagree n (%)	Unsure n (%)	Agree n (%)	Strongly Agree n (%)
*was relevant to my learning*	0 (0%)	0 (0%)	1 (2%)	42 (66%)	21 (32%)
*motivated me to apply theory and evidence to a clinical case*	0 (0%)	2 (3%)	0 (0%)	45 (72%)	16 (25%)
*motivated me to research topics beyond the material provided*	0 (0%)	3 (5%)	8 (13%)	37 (58%)	16 (25%)

*“It was a great opportunity to work up some real cases of our preferred topic.”* (P9).

*“The virtual simulation in my opinion is far more engaging than any PowerPoint lecture, as it gave me the ability to research in my own time, whilst guiding me through clinical decision making…”* (P23).

The participants also extended on the accessibility of the experience and the ease of participating in the VSIP that exposes them to the perspectives of an international expert independent of their physical location.

*"I liked that I could be at home and learn from the placement and participate. I also liked that I could raise my hand and not feel too embarrassed."* (P33).

*“We can attend from home and communicate with specialists from India”* (P20).

*“I really liked the convenience of the sessions; in that I was able to access and work through the individual content in my own time before the live session which could be viewed from the comfort of home.”* (P12).

They also expressed that the design of the VSIP offered advantages to their learning.

*“There were benefits, such as being able to hear the opinions of the other participants without any background noise, as well as the ability to look up any key concepts I didn't know about.”* (P23).

*“Benefits included being able to experience cases that we would rarely see such as ocular trauma and emergency…”* (P4).

*“Virtual simulation is more preferable as it helps in understanding the condition in depth.”* (P22).

They appreciated the role the virtual simulation played in creating an environment that they could learn in a new way.

*“I can’t see how we would’ve been able to do it face-to-face, the online environment was freeing and comfortable. I learned so much with the topics I selected and felt comfortable asking questions freely.”* (P16).

### Theme 2: the VSIP supported cognitive apprenticeship

Cognitive apprenticeship involves a master teaching an apprentice the same skill that they have mastered [12]. Through modelling and coaching, this is achieved. When discussing how they learnt through the VSIP, participants described their journey through the stages of the cognitive apprentice model beginning with the stage where a learner acquires a basic understanding of a skill or topic [13].

*“I learnt about the different cases and its management.”* (P13).

*“It was a good way to learn how to make decisions in cases like we saw in the [VSIP]. It actually gave us a very good way to view the results and how accurate do you need.”* (P5).

*“Listening to the tutors talk about how they would manage the patient was really great. I was able to compare it to my own thoughts and consider new ideas.”* (P30).

The student participants expressed that within the VSIP they sought to eliminate and correct their mistakes by making associations between different learning elements. This shows that they were also working though the associative stage of cognitive apprenticeship theory in the VSIP [13].

*“It helped me think through why I made certain choices and defend what I selected to be part of my examination. I learned the same from others and realised my own shortcomings in the cases I partook in.”* (P17).

Student participants also shared that the VSIP gave them the ability to think like the professionals and specialists. This relates to the autonomous and final stage of the cognitive apprenticeship theory [13].

*“It simulates real world [patient] with eye conditions and goes through how to diagnose and manage them. While allowing students to apply their knowledge by taking part. It’s also beneficial to have an expert on a particular condition to clarify and questions and to fill gaps in our knowledge.”* (P51).

“*I liked that I could work through things on my own, and then compare my thought processes to professionals in the field.” (*P23).

*“Getting to understand the diagnostic and clinical decision making of an ophthalmologist compared to an optometrist was interesting…we lack clarity often about what the right course of action is in our normal [problem-based learning activity]. We are often left to our often devices to understand the answer to our questions and this Virtual [Simulated] scenario was good in being clearer.”* (P4).

Discussion with the facilitator participants reiterated that the VSIP supported the various stages of cognitive parentship model.

*“… I really tried to give a lot of opportunity for the students to engage and interact…asking them what their thoughts were… I tried along the way to really discuss what my thought process was when I was going through the case, and what I had considered and what I had ruled out and the reasons for that…how [optometrists are] thinking…”* (P7).

*“… the instant feedback from the experts to see whether they're on the right track…clinical feedback that they can receive in real time… think having that feedback loop was very helpful.”* (P8).

### Theme 3: VSIP enabled clinical learning for optometric education

Researchers found student participants discussed the VSIP experience in this study as a learning activity that allows students to integrate knowledge and skills by leveraging on the simulation’s relevance to clinical practice.

*“It’s good to have an interactive session like this virtual placement which have cases that reflect real life situations. I was able to apply knowledge and also find gaps in my knowledge about certain ocular conditions.”* (P2).

They also identified that the VSIP enabled them to form links between prior learning, new learning, and the workplace.

*“…we were able to relate it to whatever case we are seeing more in the clinic and what we were observing in the clinic rather than reading books… we were easily able to relate to things.”* (P11).

Aside from the application of knowledge, researchers triangulated that the VSIP enabled a development and refinement of clinical reasoning skills from the quantitative results from the DTI-O (Table 3). A Wilcoxon Signed-Ranks test indicated that student participants self-reflected diagnostic reasoning after the VSIP was higher than before they participated in the learning activity, Z = -4.66, *p* = < 0.001. The results for the sub-domains, structured memory (Z = -3.49, *p* = < 0.001) and flexibility in thinking (Z = -3.01, *p* = 0.003), also demonstrated improvement post learning activity.

**Table 3:** Participants self-reflective diagnostic reasoning skills pre and post simulation

Category	Maximum possible score	Pre-Simulation (*n* = 46)	Post-Simulation (*n* = 46)	Mean difference (upper and lower bounds, 95% CI)
Total DTI-O	240	159.39 (±19.49)	169.47 (﻿±24.32)	10.09 (4.22–15.95)
SM DTI-O	126	76.39 (﻿±10.81)	82.41 (﻿±13.00)	6.02 (2.64–9.40)
FT DTI-O	120	80.04 (﻿±8.77)	87.04 (﻿±13.03)	6.43(3.24–9.63)

*Abbreviations*: *DTI-O* Diagnostic Thinking Inventory for Optometry, *FT* Flexibility in thinking, *SM* Structured memory

These results of the self-reflective inventory on diagnostic reasoning are also supported by the focus group discissions where participants described developing the ability to define differential diagnoses and hypotheses.

*“I developed skills in deciding a potential differential diagnosis. I learnt that it is alright to broaden the list and keep in mind particular conditions, even though they may be unlikely as there is a possibility of it occurring.”* (P3).

*“…diagnosing the differential diagnosis. That was a good thing… what if we think this and why… A clear idea to think in the right way for a particular condition."* (P4).

*“How to proceed with a case and what are all the battery of tests to be prepared and how to come at the tentative diagnosis.”* (P16).

The student participants described the virtual international placements allowed for conscious moments to breakdown the clinical learning experience in the virtual simulation.

*“I think one more skill that we learned is that rather than following a set of questions, it is to be asked in history based on the symptoms or the complaints by the patient… to rule out certain questions … which will not give us any additional information…the virtual [simulated international placement… made us realize that not all questions need to be asked, and only relevant questions could be used.”* (P1).

*“I became better at linking diagnosis and management, as well as considering how I would communicate my findings to the patient”* (P45).

Participants identified that the virtual simulation was a place to practice applying core competency skills.

*“Virtual simulation has potential, because it can introduce a continuous cycle of practice for future optometrists, so that they can become more aware of the patient experience, along with the tests and treatments required to manage this.”* (P31).

*“I think it enables us to apply knowledge more practically before we actually practice on real people”* (P18).

When student participants were asked to rate their perception of skill development through the VSIP there was an overall improvement in clinica reasoning, clinical knowledge, evidence based practice and communication (Table 4).

**Table 4:** Participants perception of clinical skill development through the virtual clinical placements

Clinical skill	Reduced a great deal n(%)	Reduced n(%)	Unsure n(%)	Improved n(%)	Improved a great deal n(%)
Clinical Reasoning	1(1%)	0 (0%)	1 (1%)	38 (64%)	20 (34%)
Clinical Knowledge	1(1%)	0 (0%)	4 (7%)	35 (58%)	20 (34%)
Evidence Based Practice	1(1%)	0 (0%)	4 (7%)	34 (56%)	21 (36%)
Communication	1(1%)	0 (0%)	16 (27%)	24 (40%)	19 (32%)

### Theme 4: VSIP’ role in cross-cultural professional identity development in optometry students

Virtual simulation can develop students’ perception of their professional identity [14]. Researchers found that student participants reflected that the VSIP were a way to interact with people from different cultures and professional environments to begin to establish cross cultural communication skills and interconnection.

The synchronous and asynchronous teaching and learning approaches embedded into the VSIP enhanced students’ understanding of the shared core values and clinical practices of the Optometric profession, along with how these values and practices are operationalized in specialized work contexts.

*“We were able to gain more information and knowledge about the work ups that they do in the specialties, both in Australia and India.”* (P21).

*"…management part in India was different so we were not allowed to prescribe or do something in India, and we are restricted when compared to the Australian scenarios. So, we were not much equipped with what is going on therapeutically."* (P3).

Additionally, the immersive nature of the virtual placements enabled students to practically apply their clinical knowledge and skills collaboratively, strengthening students’ personal, academic, and practical cross-cultural capacity and, importantly, value, knowledge, and professional socialization.

“*Interreacting people all over the world at the same time”* (P38).

*"…learn about procedures that's been followed in both Australia and in India."* (P5).

*“It was a new experience for most of us. Additionally, it was nice to be able to walk through a simulated clinical situation with the students from India. I also got a bit of understanding of how optometry is practiced in India, which slightly differs subtly from how we practice in Australia.”* (P18).

## Discussion

This mixed methods study uncovered the rich perspectives of both students and facilitators involvement in VSIP that delivered an authentic learning experience for optometry students despite the interruptions during the COVID-19 pandemic. The educational design of the IEC platform was created in a way that students and facilitators perceived the experience of being placed in a realistic clinical environment.

### Do online virtual simulation international placements in the IEC engage optometry students and motivate them to study?

Virtual simulation motivates optometry students to learn when they see relevance to their future roles and experience authentic learning activities. A previous study on optometry students’ perceptions of virtual simulation found that the correct level of realism generates enough immersion to feel as though they were performing work related training [14]. In this study participants referenced the meaningfulness of the VSIP in the IEC was supported by the authenticity in the learning experience, *“We learn optometry in a real way… rather than learning it theoretically…”* (P37). This realism reinforced the participants perception that the learning activities were relevant and motivated them to apply didactic knowledge and research beyond the curriculum.

### Do optometry students value the VSIP resources provided?

The value of the IEC virtual simulation international placements expressed by the participants was overwhelming. Firstly, the IEC allowed open collaboration between the participants. The participants also valued the accessibility of the experience and the ease of participating in a placement that exposed them to the perspectives of an international expert independent of their physical location. They repeatedly appreciated that this web-based platform could be used anywhere and at any time around the world. Importantly there were signs that the level of fidelity of the IEC was adequate because they IEC allowed them “…*the ability to look up any key concepts I didn't know about.”* (P23). This offered insight into a potential advantage for screen based virtual reality compared to high fidelity augmented reality simulators. A collective overview of optometry institutions response to adapting teaching during the Coronavirus pandemic indicated a positive shift in online teaching for using digital technologies in optometric education [15]. This paper extends their discussion with formal evaluation of emerging innovative techniques for teaching and learning that demonstrates the features that are inherent to virtual simulation support the production of inclusive international placements in future curriculum design.

In Higher Education clinical placements are used to provide students with practical experience to expediate development of clinical competency [16]. Clinical placements can be difficult to obtain, to ensure equality of experience and to provide adequate range of patient care experiences [17]. This is one of the reasons simulations, and more specifically virtual simulations, are being used to provide experience that prepare healthcare students for their future roles in clinical practice. In some fields virtual simulation is considered a potential stand in for clinical placements [17]. As virtual simulation becomes further integrated into curriculums, we need theoretical approaches to build virtual simulation pedagogy [18]. Frameworks describe the dependence of a simulation on the orchestrated fidelity and the replication or mirroring of the real work environments [19]. Others discuss the sociomaterial theoretical approaches that do not emphasise the importance on how realistic the simulation is but focus on the process of simulation that results in learning [18].

There was particular emphasis from participants that the VSIP offered a unique learning resource for each institution’s curriculum. The researchers discovered participants reflections favoured a cognitive apprenticeship learning theory. This was not the basis of the educational design but should be considered in future developments of the learning activity. The purpose of cognitive apprenticeship is to allow students to observe, practice, and enact new knowledge and skills that they’ve gleaned from a specialist [13]. This learner centred approach to learning hones in on the discrepancies found in teaching and focuses on developing mental models of cognitive and metacognitive skills, that learners need to perform at an expert-like level [12]. In the context of the virtual simulated clinical placements the student participants identified the facilitators as the experts, however future research could consider the role of the learning activity as the central axis of the cognitive apprenticeship framework. More specifically, further exploration could lead to better understanding of whether it is the experts, or the educational design of other learning activities compared to VSIP that ultimately instruct novices differently.

Additionally, there is potential for virtual clinical placement platforms such as the IEC to help students with orientation and preparation prior to attending their physical placement. If students were presented with the opportunity to virtually explore the clinical environment to familiarise themselves with the layout of the clinic and clinical staff that they are likely to work with beforehand, this would help them to feel more comfortable and prepared when it comes to attending the clinic in person. Similarly, virtual placements provide a rich and diverse range of experiences for students, as they allow students to be immersed in clinical settings around the world that they may not otherwise have a chance to visit due to safety, cost or logistical issues.

### Do VSIP in optometry produce improved optometry students’ skills and ability?

To become a graduate optometrist a holistic approach could consider not solely the level competency to be achieved but also the process, content, technologies and learning objectives with equal deliberation could prepare future optometrists to make the right decision under complex and sometimes sight threatening conditions. The virtual simulated clinical placements improved optometry participants self-perceived skills in clinical reasoning, communication, evidence-based practice, and knowledge. In addition, the results from the DTI-O before and after the learning activities showed an increase in self-perceived diagnostic reasoning ability in the context of the optometric topic. VSIP enabled students to not just learn content but experience it, growing knowledge, and skills. Further, the unique interactive, student-centered learning platform nurtured curiosity, autonomy and critical thinking, while privileging student voices, which created a learning space that catalysed considered and confident self-reflexivity, assisting students to reflect on and reconcile their professional identities.

### Do virtual international placements in optometry offer cognitive complexity that is meaningful?

Virtual mobility in this study does not replace physical mobility. However, in real life situations the opportunity to observe a patient’s clinical journey can require months before decisions can produce observable consequences. The question is, could real-life learning experiences be suboptimal to those that are carefully constructed and guides through virtual simulations? This study showed that as a virtual international placement allowed students to notice features of clinical encounters that they may not have been previously aware of. Using think aloud research in further studies may allow a richer understanding on how virtual simulations support the development of cognitive, affective, and metacognitive skills as well as identify the role the facilitator plays in supporting this through feedback and reflective prompts.

Moreover, the collaborative, cross-cultural and cross-border aspects of the virtual placement required students to be intellectually agile and consider different epistemologies in clinical reasoning and practice, particularly recognizing the intersecting dimensions of social, political and economic factors in health and wellbeing. The collaborative learning in the VSIP demonstrated similar strengths to what has been discussed in work integrated learning frameworks that support sharing stories in context of culture to students to extend of their personal professional identity development [20]. In this study, participants expressed a developing understanding that the dynamic nature of clinical practice influences professional identity due to diverse practice sites and professional demands. This increasing awareness was fostered by the design of the virtual simulation, as it offered students personal autonomy and self-regulation through self-selection of topics and asynchronous activities. These two elements, autonomy and self-regulation, are recognized as strong features that support professional identity development [21].

## Limitations

The number of participants that completed the DTI-O before the learning activity was greater than the number of students that completed the DTI-O after the activity. Although the response rate, 28%, can be considered to represent the student cohort, there was a limited ability to analyze the development and refinement of clinical reasoning skills further in terms of course stage or university enrollment.

## Conclusions

The study found that the authenticity and clinical relevance of the experience helped to motivate students to not only learn and apply didactic knowledge from the curriculum, but to also encourage them to undertake research extending beyond the content of the curriculum. The participants valued the accessibility, collaboration, and ease of participation in the IEC platform. The fidelity of the IEC platform was found to be accepted and the VSIP were considered as a potential supplement to physical clinical placements. The cognitive apprentice learning theory was identified as a possible theoretical approach for the VSIP learning activity. The study also found that the VSIP improved participants’ whilst nurturing autonomy and an awareness of global healthcare, a glimpse into professional practice and the opportunity to embody and action aspects of clinical practice. By offering future practitioners co-learning across cultures, the VSIP provide training on- demand and could revolutionize global optometric education, embedding innovative teaching and learning pedagogies in an increasingly globalized and digitized working world.

## Supplementary Information

**Additional file 1. **Student preference survey.

**Additional file 2. **Virtual Clinical Grand Rounds Placement- Student Feedback Survey.

**Additional file 3. **Focus group guide for student participants.

## Data Availability

The dataset generated and analyzed during the current study is available to the authors but is not publicly available due to ethical guidelines. The datasets used and/or analyzed during the current study available from the corresponding author on reasonable request.

## Abbreviations

IEC: International Eyecare Community
DTI-O: Diagnostic Thinking Inventor for Optometry
FT: Flexibility in thinking
SM: Structured memory
±SDs: ± Standard Deviations
