# Supplementary material for: Virtual simulated international placements as an innovation for internationalisation in undergraduate programs: a mixed methods study
Source: BMC Med Educ. 2023 Apr 19;23:258. doi: 10.1186/s12909-023-04260-x (PMC10112994; doi:10.1186/s12909-023-04260-x)
Supplement: Supplementary file 1 — Additional file 1. Student preference survey. [file 12909_2023_4260_MOESM1_ESM.pdf]

Additional file 1

Virtual simulated international placements as an innovation for internationalisation in undergraduate programs: a mixed methods study

Authors: Amanda K Edgar[1] MOptom AFANZAHPE, James A Armitage[1] PhD FAAO FACO, Nadeeka Arambewela-Colley[3] MICD, Luke X Chong[1] PhD FAAO FACO, Anuradha Narayanan[2] PhD FAAO

[1]School of Medicine (Optometry), Faculty of Health, Deakin University, 75 Pigdons Road, Waurn Ponds, Australia 3216

[2]Elite School of Optometry, Medical Research Foundation, Chennai, India

[3] Partnerships and Engagement, Office of the Executive Dean, Faculty of Health, Deakin University, 221 Burwood Highway, Burwood, Australia 3125

Address for correspondence: Amanda Edgar, School of Medicine (Optometry), Deakin University, 75 Pigdons Road, Waurn Ponds, Australia 3216

amanda.edgar@deakin.edu.au

Student preference survey

Virtual International Placements

In January - February 2021 you will have Virtual International Placements hosted by Deakin University, Australia and Elite School of Optometry - Sankara Nethralaya Eye Hospital.

Virtual International Placements will allow you to experience cross-border collaboration with optometry students, optometrists and ophthalmologists from different backgrounds and cultures to exchange knowledge and enhance intercultural understanding.

The placement sessions will be topic-specific and we are giving you the opportunity to choose two topics you would like to attend. We are asking you to choose one topic you feel confident about and another topic that you feel would be challenging. This means you will have the chance to attend a placement session focused on a topic that interests you the most, and to attend one that you feel you need the most support.

Please enter your full name here

Please choose your location

- O ESO - Elite School of Optometry
- O Deakin University

Knowing that you will have access to all content eventually, please select **one** topic that you feel your learning needs the most support

	I would like to attend
Paediatrics	0
Binocular Vision	0
Ocular Emergency	0
Contact Lens	0
Glaucoma	0
Retina	0
Low Vision	0
Neuro-ophthalmology	0
Uvea	0
Oculoplasty	0
Cornea	0

Knowing that you will have access to all content eventually, please select **one** topic that you are interested in

	I would like to attend
Paediatrics	0
Binocular Vision	0
Ocular Emergency	0
Contact Lens	0
Glaucoma	0
Retina	0
Low Vision	0
Neuro-ophthalmology	0
Uvea	0
Oculoplasty	0
Cornea	0
