# Supplementary material for: Virtual simulated international placements as an innovation for internationalisation in undergraduate programs: a mixed methods study
Source: BMC Med Educ. 2023 Apr 19;23:258. doi: 10.1186/s12909-023-04260-x (PMC10112994; doi:10.1186/s12909-023-04260-x)
Supplement: Supplementary file 2 — Additional file 2. Virtual Clinical Grand Rounds Placement- Student Feedback Survey. [file 12909_2023_4260_MOESM2_ESM.pdf]

Additional file 2

Virtual simulated international placements as an innovation for internationalisation in undergraduate programs: a mixed methods study

Authors: Amanda K Edgar[1] MOptom AFANZAHPE, James A Armitage[1] PhD FAAO FACO, Nadeeka Arambewela-Colley[3] MICD, Luke X Chong[1] PhD FAAO FACO, Anuradha Narayanan[2] PhD FAAO

[1]School of Medicine (Optometry), Faculty of Health, Deakin University, 75 Pigdons Road, Waurn Ponds, Australia 3216

[2]Elite School of Optometry, Medical Research Foundation, Chennai, India

[3] Partnerships and Engagement, Office of the Executive Dean, Faculty of Health, Deakin University, 221 Burwood Highway, Burwood, Australia 3125

Address for correspondence: Amanda Edgar, School of Medicine (Optometry), Deakin University, 75 Pigdons Road, Waurn Ponds, Australia 3216

amanda.edgar@deakin.edu.au

Virtual Clinical Grand Rounds Placement- Student Feedback Survey

In the virtual clinical grand rounds we gave you the opportunity to choose two topics, one you are interested in and one you find challenging. You then had the opportunity to complete two self-regulated learning activities and participate in a virtual clinical grand rounds placement in collaboration with Deakin University and Elite School of Optometry. This survey asks a series of questions designed to explore your experience.

Q1. What university are you attending?

- ○ ESO
- ○ Deakin University

Q2. What stage of your studies are you completing?

2nd year Bachelor of Vision Science/ Optometry	3rd year Bachelor of Optometry	4th year Bachelor of Optometry	5th year Master of optometry	6th year Master of optometry
○	○	○	○	○

Q3.

Were there benefits to hosting the virtual clinical grand rounds in the virtual simulated environment?

Q4.

Did you find any challenges or disadvantages in using the virtual simulated environment?

Q5. How accurately does the virtual simulated enviroment represent your perception of an optometry setting?

Very inaccurate	Not accurate	Unsure	Accurate	Very Accurate
○	○	○	○	○

Q6. Reflecting on the virtual simulated environment of the international eye care community please answer the following questions.

	Strongly Agree	Agree	Unsure	Disagree	Strongly Disagree
I think that I would like to use this system frequently	○	○	○	○	○

	Strongly Agree	Agree	Unsure	Disagree	Strongly Disagree
I found the system unnecessarily complex	◯	◯	◯	◯	◯
I thought the system was easy to use	◯	◯	◯	◯	◯
I think that I would need the support of a technical person to be able to use this system	◯	◯	◯	◯	◯
I found the various functions in the system were well integrated	◯	◯	◯	◯	◯
I thought there was too much inconsistency in this system	◯	◯	◯	◯	◯
I would imagine that most people would learn to use this system very quickly	◯	◯	◯	◯	◯
I found the system very awkward to use	◯	◯	◯	◯	◯
I felt very confident using the system	◯	◯	◯	◯	◯
I needed to learn a lot of things before I could get going with this system	◯	◯	◯	◯	◯

Q7.

The virtual clinical grand rounds placement:

	Strongly disagree	Disagree	Unsure	Agree	Strong[illegible] agre[illegible]
was realistic	◯	◯	◯	◯	◯
was relevant to my learning	◯	◯	◯	◯	◯
motivated me to apply theory and evidence to a clinical case	◯	◯	◯	◯	◯

	Strongly disagree	Disagree	Unsure	Agree	Stronç agre
motivated me to research topics beyond the material provided	◯	◯	◯	◯	◯

Q8.

I would prefer virtual simulation instead of a regular lecture (e.g. PowerPoint presentation)

Never	Sometimes	Unsure	Mostly	Always
◯	◯	◯	◯	◯

Q8.1. Please explain your answer

Q9.

What skills did you begin developing through the virtual clinical grand rounds experience?

→
