# Supplementary material for: Virtual simulated international placements as an innovation for internationalisation in undergraduate programs: a mixed methods study
Source: BMC Med Educ. 2023 Apr 19;23:258. doi: 10.1186/s12909-023-04260-x (PMC10112994; doi:10.1186/s12909-023-04260-x)
Supplement: Supplementary file 3 — Additional file 3. Focus group guide for student participants. [file 12909_2023_4260_MOESM3_ESM.pdf]

Additional file 3

Virtual simulated international placements as an innovation for internationalisation in undergraduate programs: a mixed methods study

Authors: Amanda K Edgar[1] MOptom AFANZAHPE, James A Armitage[1] PhD FAAO FACO, Nadeeka Arambewela-Colley[3] MICD, Luke X Chong[1] PhD FAAO FACO, Anuradha Narayanan[2] PhD FAAO

[1]School of Medicine (Optometry), Faculty of Health, Deakin University, 75 Pigdons Road, Waurn Ponds, Australia 3216

[2]Elite School of Optometry, Medical Research Foundation, Chennai, India

[3] Partnerships and Engagement, Office of the Executive Dean, Faculty of Health, Deakin University, 221 Burwood Highway, Burwood, Australia 3125

Address for correspondence: Amanda Edgar, School of Medicine (Optometry), Deakin University, 75 Pigdons Road, Waurn Ponds, Australia 3216

amanda.edgar@deakin.edu.au

Additional file 3

Focus group guide for student participants.

1. Often at University you are allocated at random to activities such as clinical placements. What was the impact of being able to self-select the field of optometry you would attend virtually? (Knowing all content would be available at a later date)

2. Reflecting back on all of the learning opportunities involved in the Virtual Clinical Grand Rounds what activities or exercises have been the most useful in developing your skills as an optometrist?
    a. Did you have a a method that helped you learn?
    b. Has this shaped your understanding of culturally responsive clinical practice?
    c. How much time did you spend on the activities before the virtual placement and after the virtual placement?
    d. What was missing?

3. Reflecting back on all the learning activities in the Virtual Clinical Grand Rounds do you remember what specific skills you felt you were developing?
    a. What aspects helped?
    b. What aspects were challenging?

4. Knowing that this was a real case, based in a real eye hospital do you think learning through virtual simulated patient encounters like this makes what you are learning meaningful?

5. At times these activities were deliberately vague or challenging. Describe the similarities or differences you experienced in making decisions in the virtual environment compared to a lived experience.
    a. What advantages or disadvantages were there performing these activities virtually?
    b. Will the virtual experience influence how you will engage with patients in the future?

Focus group guide for facilitator participants.

1. What University are you affiliated with?
    - Deakin University
    - Elite School of Optometry/Sankara Nethralaya

2. What is your scope of practice?
    - Optometrist
    - Ophthalmologist
    - Academic

<u>Interview questions for focus group</u>

3. Reflecting back on all the learning opportunities involved in the Virtual Clinical Grand Rounds what activities or exercises have been the most useful in developing students' skills as an optometrist?

   a. What skills were being developed?

4. What do you think was the impact of the virtual simulated cases being based on real patients in a real eye hospital?
   a. Was it a worthwhile experience for the students?

5. Do you see value in virtual simulation for optometric education in the future?

6. Considering everyone who participated in the virtual clinical grand rounds(students, optometrists etc…) what were the benefits or disadvantages of being involved?
